# Downregulated expression of microRNA-124 in pediatric intestinal failure patients modulates macrophages activation by inhibiting STAT3 and AChE

**DOI:** 10.1038/cddis.2016.426

**Published:** 2016-12-15

**Authors:** Yong-Tao Xiao, Jun Wang, Wei Lu, Yi Cao, Wei Cai

**Affiliations:** 1 Department of Pediatric Surgery, Xin Hua Hospital, School of Medicine, Shanghai Jiao Tong University, Shanghai, China; 2 Shanghai Institute for Pediatric Research, Shanghai, China; 3 Shanghai Key Laboratory of Pediatric Gastroenterology and Nutrition, Shanghai, China

## Abstract

Intestinal inflammation plays a critical role in the pathogenesis of intestinal failure (IF). The macrophages are essential to maintain the intestinal homeostasis. However, the underlying mechanisms of intestinal macrophages activation remain poorly understood. Since microRNAs (miRNAs) have pivotal roles in regulation of immune responses, here we aimed to investigate the role of miR-124 in the activation of intestinal macrophages. In this study, we showed that the intestinal macrophages increased in pediatric IF patients and resulted in the induction of interleukin-6 (IL-6) and tumor necrosis factor-*α* (TNF-*α*). The miRNA fluorescence *in situ* hybridization analysis showed that the expression of miR-124 significantly reduced in intestinal macrophages in IF patients. Overexpression of miR-124 was sufficient to inhibit intestinal macrophages activation by attenuating production of IL-6 and TNF-*α*. Further studies showed that miR-124 could directly target the 3′-untranslated region of both signal transducer and activator of transcription 3 (STAT3) and acetylcholinesterase (AChE) mRNAs, and suppress their protein expressions. The AChE potentially negates the cholinergic anti-inflammatory signal by hydrolyzing the acetylcholine. We here showed that intestinal macrophages increasingly expressed the AChE and STAT3 in IF patients when compared with controls. The inhibitors against to STAT3 and AChE significantly suppressed the lipopolysaccharides-induced IL-6 and TNF-*α* production in macrophages. Taken together, these findings highlight an important role for miR-124 in the regulation of intestinal macrophages activation, and suggest a potential application of miR-124 in pediatric IF treatment regarding as suppressing intestinal inflammation.

Pediatric intestinal failure (IF) is a devastating condition that can be defined as the inability to maintain sufficient nutrient, fluid, electrolyte and micronutrient, causing unsustain adequate growth in children.^1^ There are several etiologies can cause IF, mainly including intestinal atresia, necrotizing enterocolitis, hirschsprung disease and intestinal pseudo-obstruction.^2^ Although the advances in neonatal intensive care, anesthesia, nutrition support and surgical techniques have improved survival of children with IF, the severe intestinal inflammation limits consequences of therapeutic intervention.^3^

MicroRNAs (miRNAs) are evolutionarily conserved non-coding RNA oligonucleotides that can regulate the expression of numerous gene targets and have been involved in several human diseases.^4, 5^ Several recent studies have been demonstrated that miRNAs are important mediators in the pathogenesis of human inflammatory diseases.^6, 7, 8, 9, 10^ It has been identified that the signal transducer and activator of transcription 3 (STAT3) activities, a major factor in inflammatory response, are regulated by several miRNAs such as miR-21, miR-181b and miR-124.^8, 10, 11^

The vagus nerve of the autonomic nerve system has been reported to play an important role in the regulation of the inflammatory response.^12^ The acetylcholine (ACh) secreted from vagus nerve acts on tissue macrophages and inhibits the production of pro-inflammatory cytokines.^13, 14^ The Ach can bind nicotinic receptors on macrophages, intercepts the nuclear translocation of nuclear factor kappa-light-chain-enhancer of activated B cells (NF-*κ*B) and inhibits the production of pro-inflammatory mediators.^14, 15^ Acetylcholinesterase (AChE) is a main enzyme that can hydrolyze ACh. Thus, high amounts of AChE may negatively regulate the cholinergic anti-inflammatory signal.^16^ Iftach *et al.* have showed that miRNA-132 potentiates cholinergic anti-inflammatory signaling by targeting AChE.^17^ We therefore predicted that miR-124 can attenuate intestinal macrophages activation by targeting both STAT3 and AChE.

## Results

### The intestinal macrophages increased in pediatric intestinal failure patients

**Figure 1:**
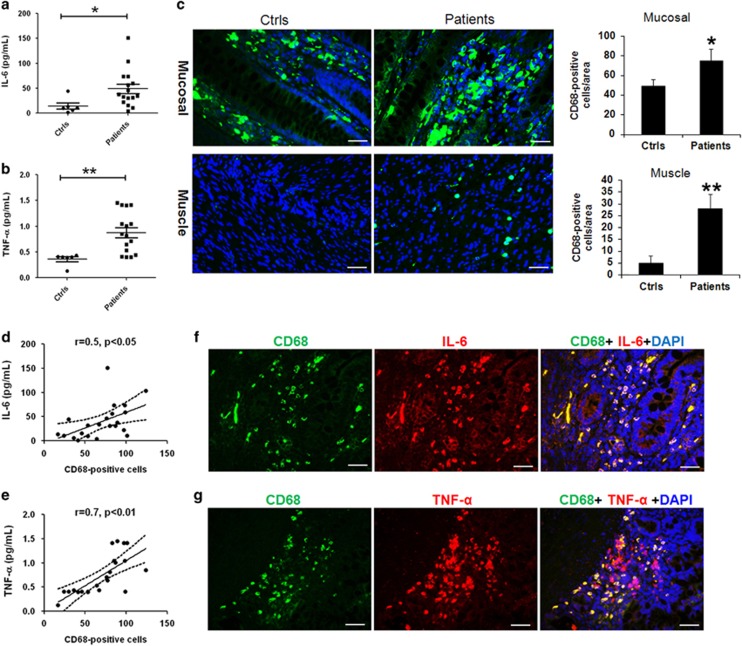
Intestinal macrophages increased in pediatric IF patients. (**a** and **b**) The levels of IL-6 and TNF-*α* in serum were significantly higher in patients (*n*=16) than in controls (*n*=20). (**c**) The number of CD68-positive macrophages increased in both mucosal and muscle layers from patients (controls, *n*=6, patients, *n*=16). (**d** and **e**) The numbers of CD68-positive macrophages were positively associated with the concentrations of IL-6 and TNF-*α* in serum. (**f** and **g**) The proteins of IL-6 and TNF-*α* were localized in the CD68-positive macrophages in patients (*n*=16). Scale bar=50 *μ*m. *P<0.05; **P<0.01

A total of 16 pediatric patients with IF participated in the study. Causes of IF included necrotizing enterocolitis (*n*=3), small bowel atresia (*n*=3), mid-gut volvulus (*n*=3), chronic intestinal pseudo-obstruction (*n*=4) and aganglionosis of hirschsprung's disease (*n*=3). Six normal intestinal tissues that were taken from the choledochal cyst patients used as controls (Supplementary Table 1). As shown in Figure 1, it showed that the levels of pro-inflammatory cytokines IL-6 and TNF-*α* in serum were significantly higher in patients compared with controls (Figures 1a and b). Since the macrophages playing an important role in the pro-inflammatory cytokines production,^18^ we performed immunofluorescence analysis to identify macrophages populations in the intestinal mucosal and muscular layers with a pan-macrophage marker CD68. We found that the mucosal density of CD68-positive macrophages was significantly higher in patients compared with pediatric controls (Figure 1c). Notably, many CD68-positive macrophages were found in the muscle layer, in contrast to those in the muscle layer of controls (Figure 1c and Supplementary Figure 1). Correlated analysis showed that intestinal CD68-positive macrophages were positively correlated with both the levels of IL-6 (*r*=0.5, *P*<0.05) and TNF-*α* (*r*=0.7, *P*<0.01) (Figures 1d and e). In addition, we showed TNF-*α* and IL-6 co-localized in CD68-positive cells, indicating the intestinal macrophages are the major source of IL-6 and TNF-*α* released in patients (Figures 1f and g).

### The miR-124 expression reduced in intestinal macrophages of pediatric intestinal failure patients

**Figure 2:**
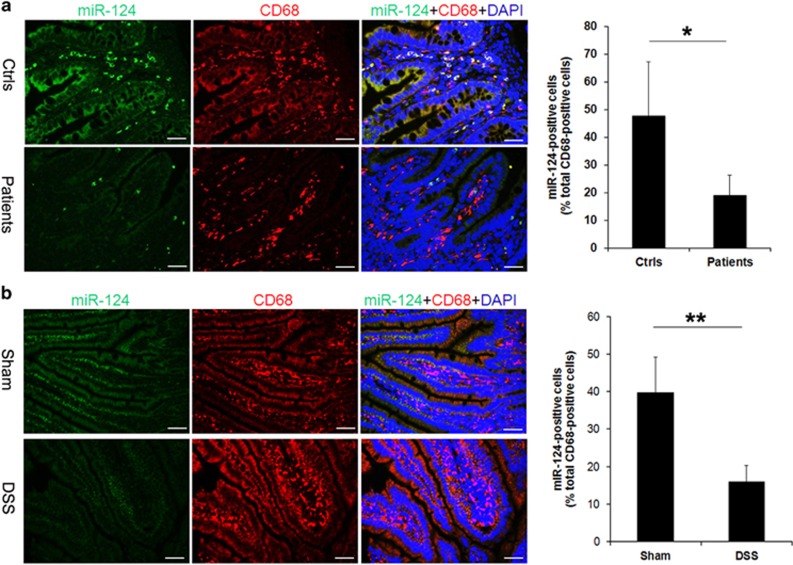
miR-124 expression decreased in intestinal macrophages of IF patients and DSS-treated mice. (**a**) FISH combined IF analysis showed that miR-124 expression was lower in patients' intestinal macrophages than in controls (controls, *n*=6, patients, *n*=16). (**b**) The miR-124 decreased in CD68-positive cells in DSS-treated mice (Sham group, *n*=6, DSS group, *n*=8). Scale bar=50 *μ*m. *P<0.05; **P<0.01

To detect the expression of miR-124 in intestinal macrophages, intestinal biopsies were processed for miR-124 fluorescence *in situ* hybridization (FISH) and CD68 immunofluorescence co-staining. As shown in Figure 2, we showed that among CD68-positive macrophages detected in the mucosal layer, the proportion expressed miR-124 was very minor and significantly lower in patients when compared with controls (Figure 2a and Supplementary Figure 2). In order to confirm this observation, we established mice model of colitis using dextran sulfate sodium (DSS). Similarly, we showed that miR-124 expression in CD68-positive macrophages reduced significantly DSS mice compared with sham ones (Figure 2b).

### The expression p-STAT3 and AChE increased in intestinal macrophages of pediatric intestinal failure patients

**Figure 3:**
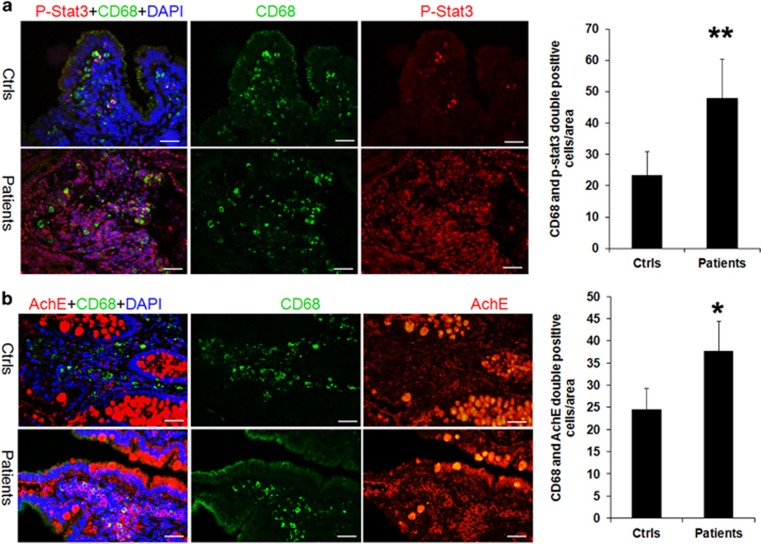
The expression of p-STAT3 and AChE increased in intestinal macrophages of IF patients. (**a**) Double staining analysis showed that p-STAT3 and AChE increasingly expressed in CD68-positive macrophages in pediatric IF patients (*n*=16), relative to controls (*n*=6). Scale bar=50 μm. *P<0.05; **P<0.01

Since the STAT3 and AChE are important mediators in inflammation response, we here examined the levels of phosphorylated STAT3 (p-STAT3) at tyrosine 705 (Tyr705) and AChE in pediatric IF patients and controls using immunohistochemistry analysis. We showed that the phosphorylation levels of STAT3 as well as AChE were significantly increased in intestinal biopsies derived from patients, relative to controls (Supplementary Figure 3). To assess the contrition of STAT3 and AChE in the intestinal macrophages activation, we detected the expression of STAT3 and AChE in macrophages with co-staining with CD68, p-STAT3 or AChE. The double immunofluorescence analysis showed that minor populations of p-STAT3-positive mucosal resident macrophages in the controls. In contrast, the numbers of cells that double-positive with CD68 and p-STAT3 significantly increased in patients (Figure 3a). Similarly, the number of macrophages that expressed AChE increased significantly in patients compared with controls (Figure 3b and Supplementary Figure 4). In the model of mice colitis, we also found that p-STAT3 and AChE increased expressed in intestinal CD68-positive cells in DSS mice compared with sham ones (Supplementary Figure 5). Thus, it suggests that the activities of STAT3 and AChE may play an important role in the macrophages activation.

### miR-124 represses the STAT3 and AChE through directly binding their 3′-untranslated regions

**Figure 4:**
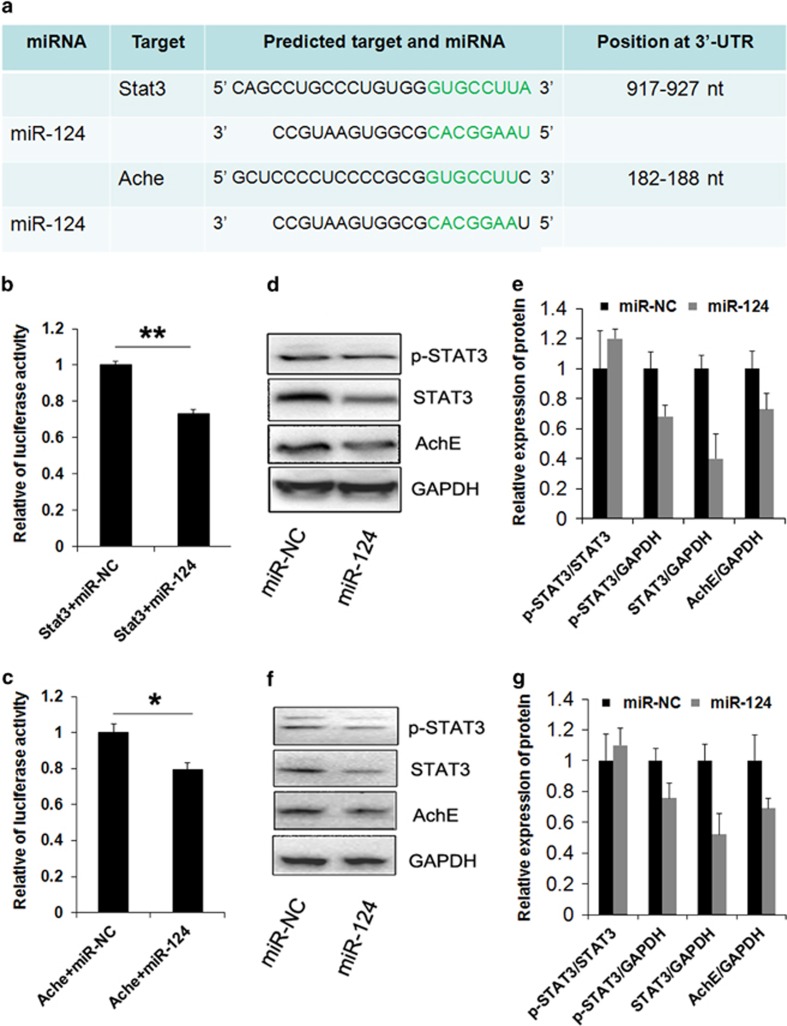
miR-124 targets STAT3 and AChE. (**a**) Bioinformatic prediction of miR-124 binding sites in the 3′-UTR of human STAT3 and AChE genes. (**b** and **c**) Relative luciferase activity of reporter constructs containing the 3′-UTR of STAT3 and AChE. Western blot analysis of STAT3 and AChE expression in THP-1-derived macrophages (**d** and **e**) and in RAW264.7 cells (**f** and **g**) transfected with miR-124. *P<0.05; **P<0.01

Using TargetScan (http://www.targetscan.org) to predict targets of miR-124, we found that the 3′-untranslated region (3′-UTR) of STAT3 mRNA and AChE mRNA contain putative miR-124 target sites (Figure 4a). To determine whether the STAT3 mRNA and AChE mRNA are potentially regulated by miR-124, we constructed reporter plasmids by inserting the cDNAs corresponding to the 3′-UTRs of mouse STAT3 mRNA and AChE mRNA into the renilla/luciferase reporter plasmids. For cells transfected with the reporter plasmid containing 3′-UTR of STAT3, the miR-124 mimic significantly suppressed luciferase activity (Figure 4b). Similarly, miR-124 mimics also significantly decreased luciferase activity in cells transfected with reporter constructs that contain the 3′-UTR of AChE (Figure 4c). The effects of miR-124 on STAT3 or AChE were then analyzed in mice macrophages RAW264.7 and human THP-1-derived macrophages. As shown in Figure 4, the levels of STAT3 decreased significantly in the cells were transfected with the miR-124 mimic for 36 h. Furthermore, transfection of the miR-124 mimic also resulted in a significant decrease in the p-STAT3 (Tyr705) (Figure 4d–g). We also showed that miR-124 evidently reduced the protein levels of AChE relative to the control miRNA treatment (Figure 4d–g).

### miR-124, STAT3 inhibitor and AChE inhibitor inhibit macrophages to release the TNF-*α* and IL-6

**Figure 5:**
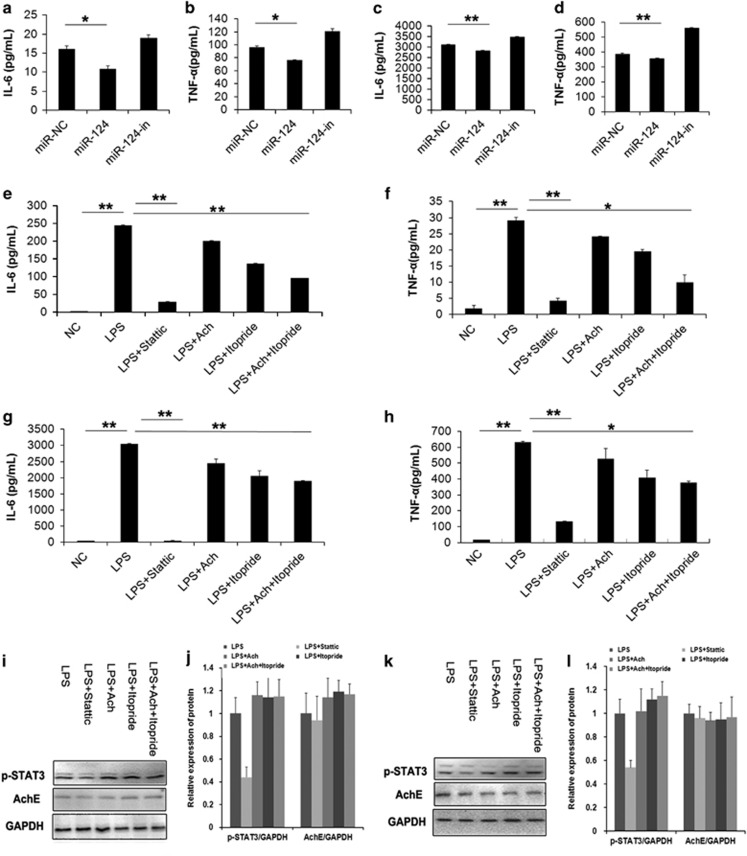
miR-124 attenuates the IL-6 and TNF-*α* production in macrophages. ELISA assay showed that overexpression of miR-124 significantly suppressed the LPS-induced IL-6 and TNF-*α* production in macrophages. (**a** and **b**) The human macrophages derived from THP-1 cells. (**c** and **d**) The mice macrophages RAW264.7. The static, Ach and itopride inhibited the IL-6 and TNF-*α* production in macrophages. (**e** and **f**) The human macrophages induced from THP-1 cells. (**g** and **h**) The RAW264.7 cells. (**i**-**l**) The expression of p-STAT3 and AChE proteins. *P<0.05; **P<0.01

To directly assess the effect of miR-124 on the production of pro-inflammatory cytokines, miR-124 was overexpressed in RAW264.7 cells and human THP-1-derived macrophages with miR-124 mimics or inhibitors transfection. As shown in Figure 5, overexpression of the miR-124 resulted in a significant reduction in lipopolysaccharide (LPS)-stimulated IL-6 and TNF-*α* production. Contrary to miR-124 overexpression, miR-124 deficiency with its inhibitors transfection increased the level of IL-6 and TNF-*α* in the supernatant compared with controls. It means that miR-124 negate the macrophages activation induced by LPS. To investigate the effects of STAT3 and AChE on the production of TNF-*α* and IL-6, RAW264.7 macrophages and human THP-1-derived macrophages were pre-incubated with static, acetylcholine (Ach), itopride or Ach plus itopride 1 h before LPS stimulated for 6 h and the cytokine responses were measured by enzyme-linked immunosorbent assay (ELISA). Stattic is a small-molecule inhibitor of STAT3 activation, which selectively inhibits tyrosine phosphorylation of STAT3.^19, 20^ As shown in Figure 5, stattic treatment significantly inhibited LPS-induced TNF-*α* and IL-6 production (Figures 5e–l). Moreover, Ach treatment repressed the macrophages to release TNF-*α* and IL-6 (Figures 5e–l). Iitopride is an active AChE inhibitor, which exerts noncompetitive inhibition on AChE activity.^21^ We here found that Iitopride significantly inhibited LPS-mediated TNF-*α* and IL-6 production from macrophages (Figures 5e–l).

### STAT3 inhibition induces expression of miR-124 in macrophages

**Figure 6:**
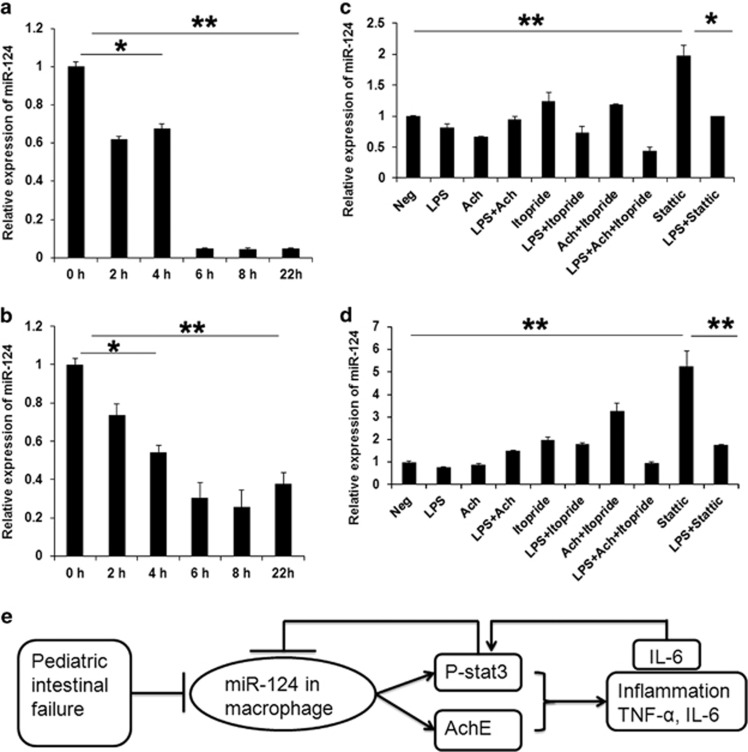
STAT3 inhibition induced miR-124 expression in macrophages. (**a** and **b**) LPS inhibited the expression of miR-124 in THP-1-derived macrophages and RAW264.7 cells at indicated time. (**c** and **d**) Stattic treatment induced miR-124 expression in THP-1-derived macrophages and RAW264.7 cells. (**e**) Scheme illustrating a potential mechanism in which miR-124 modulates the intestinal inflammation via regulating the macrophages activation. *P<0.05; **P<0.01

To determine whether LPS induces or inhibits miR-124 expression in macrophages, we stimulated RAW264.7 macrophages and human THP-1-derived macrophages with 100 ng/ml LPS for different time by quantitative PCR (qPCR). As shown in Figure 6, levels of miR-124 gradually declined 2 h after LPS addition, reached to the bottom at ~6 h (Figures 6a and b). To further test whether the STAT3 and AChE have effects on the expression of miR-124, the activities of STAT3 and AChE were blocked with static and itopride, and the levels of miR-124 were determined by qPCR after 4 h with LPS stimulation. It found that static treatment significantly increased the expression of miR-124 when compared with LPS administration (Figures 6c and d). The levels of miR-124 did not increase significantly in the cells with addition LPS plus itopride or Ach when compared with LPS treatment (Figures 6c and d).

## Discussion

In this study, we observed that the number of macrophages increased in intestinal tissues from pediatric IF patients, resulting in inflammatory cytokines TNF-*α* and IL-6 increased. We found that the levels of miR-124 were reduced in intestinal macrophages, whereas the expression of p-STAT3 and AChE increased in intestinal macrophages. In functional studies, reintroduction of miR-124 dramatically repressed macrophages activation by reducing TNF-*α* and IL-6 production. miR-124 was found to directly target the genes of STAT3 and AChE. Furthermore, STAT3 inhibition or AChE inhibtition significantly suppressed the LPS-stimulated TNF-*α* and IL-6. These findings suggest a potential application of miR-124 in pediatric IF treatment regarding as suppressing intestinal inflammation.

Intestinal macrophages localized within the lamina propria are the first phagocytic cells of the innate immune system to interact with microorganisms and microbial products that have breached the epithelium.^22, 23^ In health, intestinal macrophages do not produce pro-inflammatory cytokines.^24^ In inflammatory bowel disease, inflammatory bowel disease intestinal macrophages increase and release pro-inflammatory cytokines.^25^ In the present study, the immunofluorescence staining showed that a significant increase in CD68-positive macrophages was observed in intestinal mucosal and muscular layers from pediatric IF patients. The increased macrophages in patients were associated with the pro-inflammatory cytokines TNF-*α* and IL-6. The co-localization of CD68 with TNF-*α* and IL-6 indicated that intestinal macrophages are the major source of TNF-*α* and IL-6 released in IF patients.

There are several studies recently demonstrate that miRNAs are key modulators in the inflammatory response.^6, 10, 26, 27^ We here showed that the expression of miR-124 was reduced in intestinal macrophages of patients relative to controls. It reported that the miR-124 could target many genes when it exerting its effects.^8, 28, 29, 30, 31, 32^ Notably, miR-124 can regulate the expression of STAT3, which is an important transcriptional factor for many inflammatory cytokines.^10^ We showed that the phosphorylation levels of STAT3 at Tyr705 were significantly increased in intestinal biopsies derived from patients, relative to controls. Furthermore, the number of co-staining of phosphorylated STAT3 and CD68 cells increased significantly in patients. With informative tools, we predicted that the miR-124 potentially targeted AChE. AChE is an enzyme that mainly to hydrolyze ACh. A previous study demonstrated that ACh could inhibit the production of pro-inflammatory mediators via binding *α*7 nicotinic receptors on macrophages.^33^ Therefore, high expression of AChE could potentially negate the cholinergic anti-inflammatory signal.^16^ We showed that the expression of AChE significantly increased in intestinal macrophages of patients, suggests that AChE may play an important role in intestinal macrophages activation. On the basis of the above information, we investigated the potential direct interaction of miR-124 and STAT3, and interaction of miR-124 and AChE. Bioinformatic analysis identified sequence complementarity of miR-124 with the 3′UTR of STAT3 and AChE. To verify the interaction of them, we employed dual luciferase reporter assays. Delivery of miR-124 significantly suppressed STAT3-3′UTR and AChE-3′UTR luciferase activities. To further validate the correlation between STAT3 expression and phosphorylation status, we performed western blot analysis for total and phosphorylated STAT3 in human and mice macrophages, after miR-124 overexpression. MiR-124 overexpression suppressed both total and phosphorylated STAT3 at similar levels, suggesting that miR-124 regulates STAT3 activity through transcriptionally repressing the total STAT3 protein level. Moreover, the AChE protein was also suppressed by miR-124 delivery in these macrophages.

We further investigated whether miR-124 occurs in LPS-stimulated macrophages activation. In mice RAW267.4 macrophages and human THP-1-derived macrophages, the miR-124 overexpression attenuated the LPS-stimulated IL-6 and TNF-*α* production. In addition, the static, inhibitor of STAT3, Ach, as well as itopride, AChE inhibitor, also significantly inhibited LPS-induced TNF-*α* and IL-6 production in macrophages. Hong Wang *et al.*^34^ reported that the Ach could inhibit macrophages release TNF-*α* through binding the nicotinic acetylcholine receptor *α*7 subunit on the macrophages. We thus suggest that AChE inhibitor suppresses macrophages activation by decreasing the acetylcholine hydrolyzation and improving the cholinergic anti-inflammatory pathway. Taken together, we thought that miR-124 potentially modulated macrophages activation by targeting the STAT3 and AChE. In this study, we also found that LPS decreased the miR-124 expression in macrophages. At the same time, the increased IL-6 induced by LPS could stimulate the downstream factor STAT3. Interestingly, it showed that the STAT3 inhibition significantly induced the expression of miR-124. Thus, it suggested that downregulation of STAT3 by miR-124 appears to restrict the positive feedback, leading to a reduction in IL-6 production.

In summary, in present study, we investigated the potential role of miR-124 in intestinal inflammation and its underlying mechanisms. Our findings suggest that downregulation of miR-124 plays an important role in intestinal macrophages activation during pediatric IF development and progression, and that miR-124 could be employed as an effective therapeutic target for pediatric IF regarding as the inhibition of intestinal inflammation.

## Materials and Methods

### Tissue specimens

A total of 16 intestinal and serum specimens were retrieved from pediatric IF patients who underwent surgery. Six normal tissues that were taken from the choledochal cyst patients used as controls. Surgically removed intestinal tissues were immediately fixed in 10% neutral buffered formalin for about 24 h and go through dehydration, clearing and paraffin embedding. Sections were mounted on positively charged slides after cutting at 4 *μ*m thick, baked at 65 °C for 1 h and then stored at room temperature (RT) for later use. A total of 20 serum specimens were obtained from healthy children were used as serum controls. All patients' guardians provided written informed consents. This study was approved by the Faculty of Medicine's Ethics Committee of Xin Hua hospital, School of Medicine, Shanghai Jiao Tong University. The clinical characteristics of the patients are presented in Supplementary Table 1.

### DSS-induced colitis in mice

Male 6-weeks-old C57BL/6J mice were randomized to Sham group (*n*=6), DSS group (*n*=8). Intestinal colitis was induced by treatment of mice with 3% DSS (36–50 kDa, MP Biomedicals, Solon, OH, USA) dissolved in the drinking water for 7 days as previously described.^35^ All experiments were approved by the Animal Care Committee of Xin Hua Hospital, Shanghai Jiao Tong University.

### Immunohistochemistry analysis

Immunohistochemistry was performed using the chromogen diaminobenzidine as we described previously.^36^ Briefly, the slides were incubated with xylol and descending concentrations of ethanol. Endogenous peroxidases were blocked by using 0.3% H_2_O_2_ for 10 min at RT. After antigen retrieval, blocking was performed using 5% bovine serum albumin for 30 min at RT. The antibodies of anti-p-STAT3 and anti-AChE were applied at their optimal concentration overnight in a wet chamber for overnight at 4 °C. The slides were rinsed in phosphate-buffered saline (PBS) and incubated with the appropriate secondary antibody for 1 h at RT. Antibody binding was visualized using a liquid diaminobenzidine Substrate Chromogen System (Dako, Glostrup, Denmark). The slides were rinsed in PBS and counterstained with hematoxylin. Immunohistochemistry images analysis was used software Image Pro Plus (Media Cybernetics, Rockville, MD, USA) 10 fields/sample.

### miRNA fluorescence *in situ* hybridization

miRNA-124 FISH was performed using an existing protocol.^37^ Briefly, the sides were deparaffinized in xylene and rehydrated in serial ethanol solutions, and diethylpyrocarbonate-treated water and PBS wash. After deparaffinized step, the endogenous peroxidase was quenched with 0.024 M HCl in ethanol, and incubated with proteinase K (20 *μ*g/ml, 37 °C for 10 min) followed by a PBS wash (2 × 5 min), and end with a 2 × saline-sodium citrate buffer wash. Hybridize overnight at 50 °C with probe specific to miR-124 at the concentration of 25 nM in a moist chamber. Locked nucleic acid-based miR-124 probe was labeled with biotin at the 3′ end. The probe sequences for human and mice are 5′-TAAGGCACGCGGTGAATGCC-3′ and 5′-GGCATTCACCGCGTGCCTTA-3′. Next, the slides were washed with saline-sodium citrate and PBST (PBS containing 0.1% tween-20). Lastly, the anti-biotin labeled fluorescein isothiocyanate was to visualize the positive hybridization signals, and the signals were observed under the fluorescent microscopy (Nikon, Eclipse Ti, Tokyo, Japan).

### Immunofluorescence staining

The immunofluorescence assays were performed with the methods we described previously.^38^ Briefly, the intestinal sections were incubated with xylol and descending concentrations of ethanol. Endogenous peroxidases were removed with 0.3% H_2_O_2_. The CD68, AchE and/or p-STAT3 antibodies was then applied at an optimal concentration (CD68, 1:100, AchE, 1:100 and p-STAT3, 1:50) overnight in a wet chamber after blocking with 3% bovine serum albumin for 1 h at RT. The slides were rinsed in PBS and incubated with the secondary antibodies. After washing three times with PBS, the secondary antibody conjugated to Alexa Fluor 488 or Alexa Fluor 647 was applied to these slides for 1 h at RT. The nuclei were counterstained with 4′, 6-diamidino-2-phenylindole. The total cell number of CD68+, CD68+/p-STAT3+ and CD68+/AchE+ cells were counted from randomly selected 10 view fields for each sample.

### miRNA FISH and immunofluorescence co-staining

To assess whether miR-124 was expressed in intestinal macrophages, intestinal sections were processed for double immunofluorescence staining to visualize the simultaneous localization of miR-124 (green) and a primary antibody for CD68 (red), a known macrophage pan-marker. First, we performed FISH to detect miR-124, and then, we used immunofluorescence to label macrophages. The applying FISH and protocol immunofluorescence were described earlier. The total cell number of CD68+/miR-124+ cells was counted from randomly selected 10 view fields for each sample.

### Cell culture

RAW264.7 cells, a murine macrophage cell line, were obtained from Dr WJ. RAW264.7 cells were cultured in high-glucose DMEM (Invitrogen, Carlsbad, CA, USA) containing 10% fetal bovine serum (FBS, Invitrogen), penicillin-streptomycin (Invitrogen), in a humidified atmosphere of 5% CO_2_ at 37 °C. Human THP-1 cells were purchased from Shanghai Institute of Cell Biology (Shanghai, China) and cultured in RPMI 1640 media with 10% FBS phorbol esters (PMA) can induce this cell line to differentiate into macrophages.^39^ This was accomplished by washing and resuspending the cells in fresh medium containing 100 ng/ml PMA.^40^ After 48 h, the cells were washed three times and cultured overnight in fresh culture medium, then processed as follows for cytokine production. For experiments, the RAW264.7 cells or THP-1-derived macrophages were cultured in six-well plates and transfected with miR-124 mimics, inhibitors or miRNA controls. To determine the role of LPS, these cells were treated with 100 ng/ml LPS for 0, 2, 4, 6, 8 and 22 h. To study their effects, acetylcholine (Ach, 100 *μ*M) itopride hydrochloride (AChE inhibitor, 50 *μ*M), Ach plus itopride and stattic (STAT3 inhibitor, 10 *μ*M) were added to the medium 1 h before 100 ng/ml LPS stimulation and continued to incubation for 4 h.

### Enzyme-linked immunosorbent assay

Serum samples were stored at −80 °C until analyzed after blood collection and centrifugation. IL-6 and TNF-*α* concentrations in serum were measured by ELISA kits (R&D Systems, MN, USA) according to the manufacturer's instructions. The IL-6 and TNF-*α* concentrations in supernatants were determined by ELISA kits (eBioscience, CA, USA) followed the manufacturer's protocols.

### Dual luciferase reporter assay

The potential targets of miR-124 were predicted using targetScan software (http://genes.mit.edu/targetscan). To construct reporter vectors that contained the miR-124-target sites, we synthesized the gene fragments containing the predicted targets of the 3′-UTRs of human STAT3 and AChE and subsequently cloned them into the psiCHECK2 vectors. The 3′-UTR vectors were cotransfected with miRNA-124 or a negative control into HEK293 cells using Lipofectamine 2000. The cell lysates were collected at 72 h after transfection, and the reporter activity was measured with the Dual Luciferase Assay (Promega, Madison, WI, USA). The firefly luciferase values were normalized to renilla, and the firefly/renilla ratios are presented.

### Western blots

Western blots were performed as previously described.^1^ Cells were lysed with lysis buffer after rinsed with ice-cold PBS. Protein of 30 *μ*g/well was separated on 4–12% SDS-polyacrylamide gels and transferred onto nitrocellulose using a dry blotting system (iBlot system, Invitrogen). After blocking in PBS, 5% nonfat milk at RT for 30 min, membranes were incubated with the primary antibodies overnight at 4 °C. The membranes were washed three times with PBS contained 0.1% Tween-20, and then incubated with secondary antibodies. The signals were detected using ECL chemiluminescence reagents. (Pierce, Rockford, lL, USA). The primary antibodies of phosphorylated STAT3, total STAT3, AChE and GAPDH were performed in this study.

### miRNA real-time PCR

Total RNA was extracted from cells using Trizol (Invitrogen) according to the protocol of the manufacture. miRNAs were reversed transcribed using miRNA-specific primers (Shanghai GenePharma, Shanghai, China). Subsequently, real-time PCR reactions were performed using the PikoReal Real-Time PCR System (The Thermo Scientific, Waltham, MA, USA) with SYBR green mixture (Applied Biosystems, Foster City, CA, USA). PCR reactions were incubated in a 96-well plate at 95°C for 3 min, followed by 40 cycles at 95 °C for 15 s and 62 °C for 1 min. All samples were assayed in triplicate, and data were normalized to endogenous RNU6B. Relative miRNA expression levels were calculated using the ΔΔCt method.

### Statistical analyses

All data are reported as the mean±S.D. For comparisons of different groups, statistical significance was determined based on the Student's *t*-test or ANOVA analysis. *P*-values <0.05 were considered to be statistically significant. For additional 'Materials and Methods', please refer to the Supplementary data.
