# Supplementary material for: Downregulated expression of microRNA-124 in pediatric intestinal failure patients modulates macrophages activation by inhibiting STAT3 and AChE
Source: Cell Death Dis. 2016 Dec 15;7(12):e2521–. doi: 10.1038/cddis.2016.426 (PMC5260981; doi:10.1038/cddis.2016.426)
Supplement: Supplementary Information [file cddis2016426x1.doc]

**Supplemental Tables and Figures**

**Downregulated expression of microRNA-124 in pediatric intestinal failure patients modulates macrophages activation by inhibiting STAT3 and AChE**

Yong-Tao Xiao^1,2,3^, Jun Wang^1,3^ , Wei Lu ^1^, Yi Cao^1,3^, Wei Cai ^1,2,3^

**Table 1. Patients characteristics**

| **Groups** | **Age** | **Gender** | **Diagnosis** | **Tissues** |
| --- | --- | --- | --- | --- |
| **Intestinal failure** | Male | 3m | necrotizing enterocolitis | Jejunum, ileum |
| **Intestinal failure** | Female | 4m | necrotizing enterocolitis | Jejunum, ileum |
| **Intestinal failure** | Male | 54d | necrotizing enterocolitis | Jejunum |
| **Intestinal failure** | Female | 6m | chronic intestinal pseudo-obstruction | Jejunum ileum |
| **Intestinal failure** | Female | 2m | chronic intestinal pseudo-obstruction | Jejunum |
| **Intestinal failure** | Female | 1y | chronic intestinal pseudo-obstruction | Jejunum ileum |
| **Intestinal failure** | Female | 27d | chronic intestinal pseudo-obstruction | Jejunum |
| **Intestinal failure** | Male | 1y | mid-gut volvulus | Jejunum |
| **Intestinal failure** | Female | 1y | mid-gut volvulus | Jejunum, ileum |
| **Intestinal failure** | Female | 2y | mid-gut volvulus | Jejunum ileum |
| **Intestinal failure** | Male | 3y | small bowel atresia | Jejunum ,ileum |
| **Intestinal failure** | Female | 4m | small bowel atresia | Jejunum |
| **Intestinal failure** | Male | 2y | small bowel atresia | Jejunum ileum |
| **Intestinal failure** | Female | 2y | aganglionosis of Hirschsprung’s disease | Jejunum |
| **Intestinal failure** | Female | 3y | aganglionosis of Hirschsprung’s disease | Jejunum, ileum |
| **Intestinal failure** | Female | 5m | aganglionosis of Hirschsprung’s disease | Jejunum, ileum |
| **Control** | Female | 2m | choledochal cyst | Jejunum |
| **Control** | Male | 2y | choledochal cyst | Jejunum |
| **Control** | Male | 15m | choledochal cyst | Jejunum |
| **Control** | Male | 31d | choledochal cyst | Jejunum |
| **Control** | Male | 15m | choledochal cyst | Jejunum |
| **Control** | Female | 1y | choledochal cyst | Jejunum |

**Figure 1. The CD68-positive cells in the intestinal tissues of intestinal failure patients from the each patient etiology.** Controls (ctrls, n=6), necrotizing enterocolitis (NEC, n=3), small bowel atresia (n=3), mid-gut volvulus (n=3), chronic intestinal pseudo-obstruction (CIPO, n=4) and aganglionosis of hirschsprung’s disease (HD, n=3). Scale bar = 50 μm.


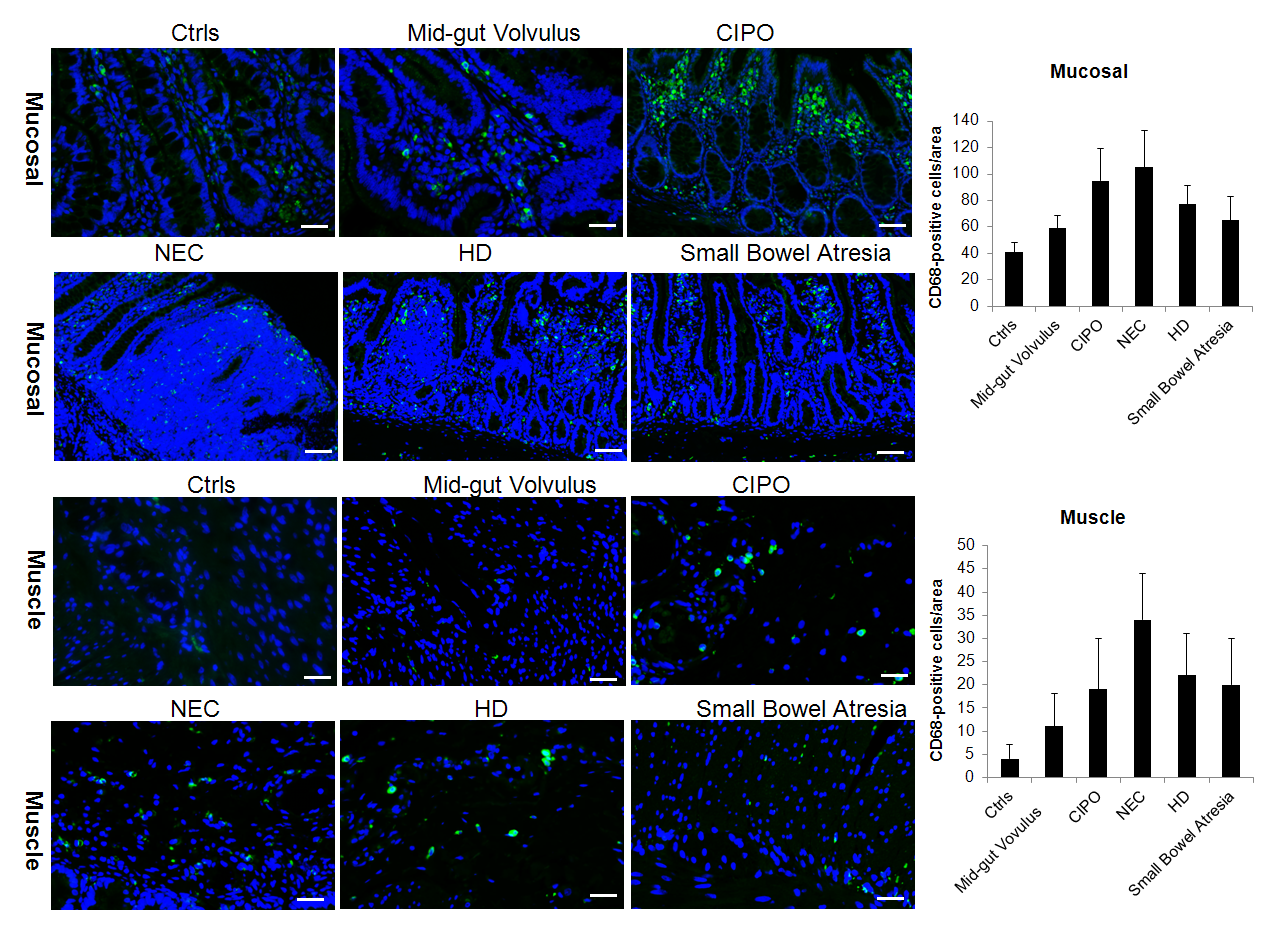


**Figure 2. miR-124 expressed in intestinal macrophages from the each patient etiology.** Controls (ctrls, n=6), necrotizing enterocolitis (NEC, n=3), small bowel atresia (n=3), mid-gut volvulus (n=3), chronic intestinal pseudo-obstruction (CIPO, n=4) and aganglionosis of hirschsprung’s disease (HD, n=3). Scale bar = 50 μm.

**
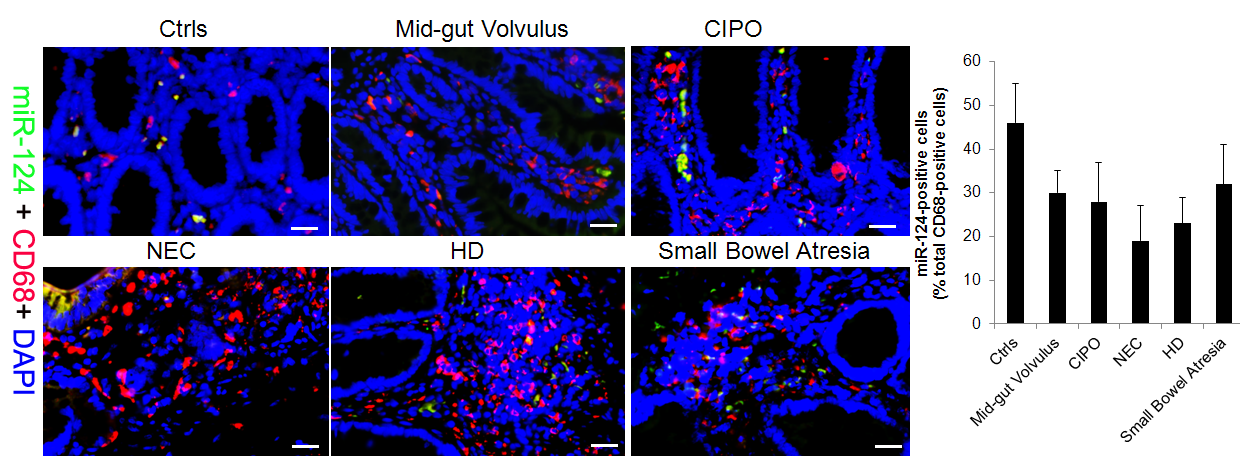
**

**Figure 3. The expression of p-STAT3 and AChE was analyzed in intestinal failure patients from the each patient etiology.** Controls (ctrls, n=6), necrotizing enterocolitis (NEC, n=3), small bowel atresia (n=3), mid-gut volvulus (n=3), chronic intestinal pseudo-obstruction (CIPO, n=4) and aganglionosis of hirschsprung’s disease (HD, n=3). IHC images analysis was used software Image Pro Plus (Media Cybernetics) 10 fields/sample. Scale bar = 50 μm.

**
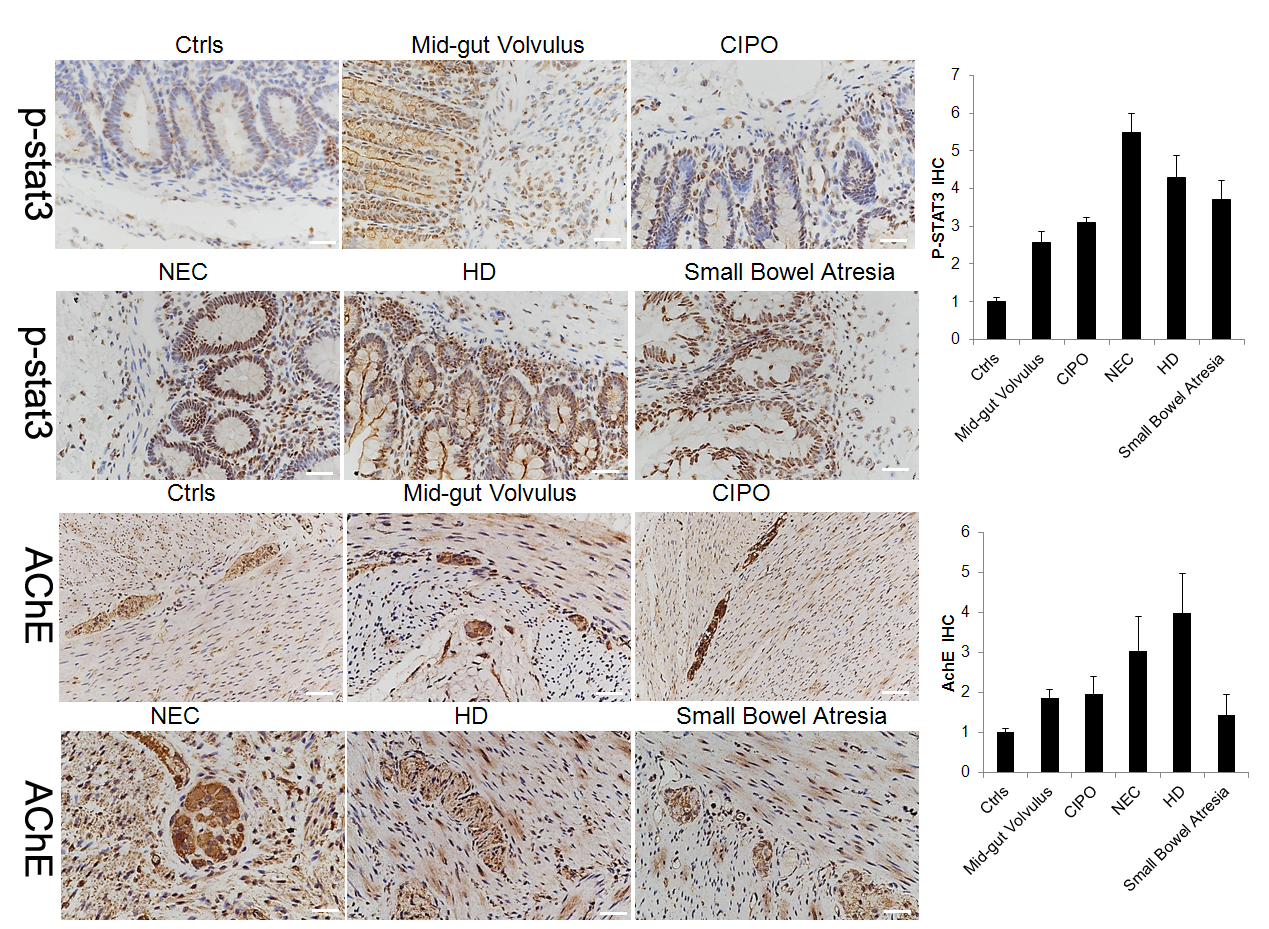
**

**Figure 4. The expression of p-STAT3 and AChE in pedatric intestinal failure patients’ intestinal macrophages.** Controls (ctrls, n=6), necrotizing enterocolitis (NEC, n=3), small bowel atresia (n=3), mid-gut volvulus (n=3), chronic intestinal pseudo-obstruction (CIPO, n=4) and aganglionosis of hirschsprung’s disease (HD, n=3). Scale bar = 50 μm.

**
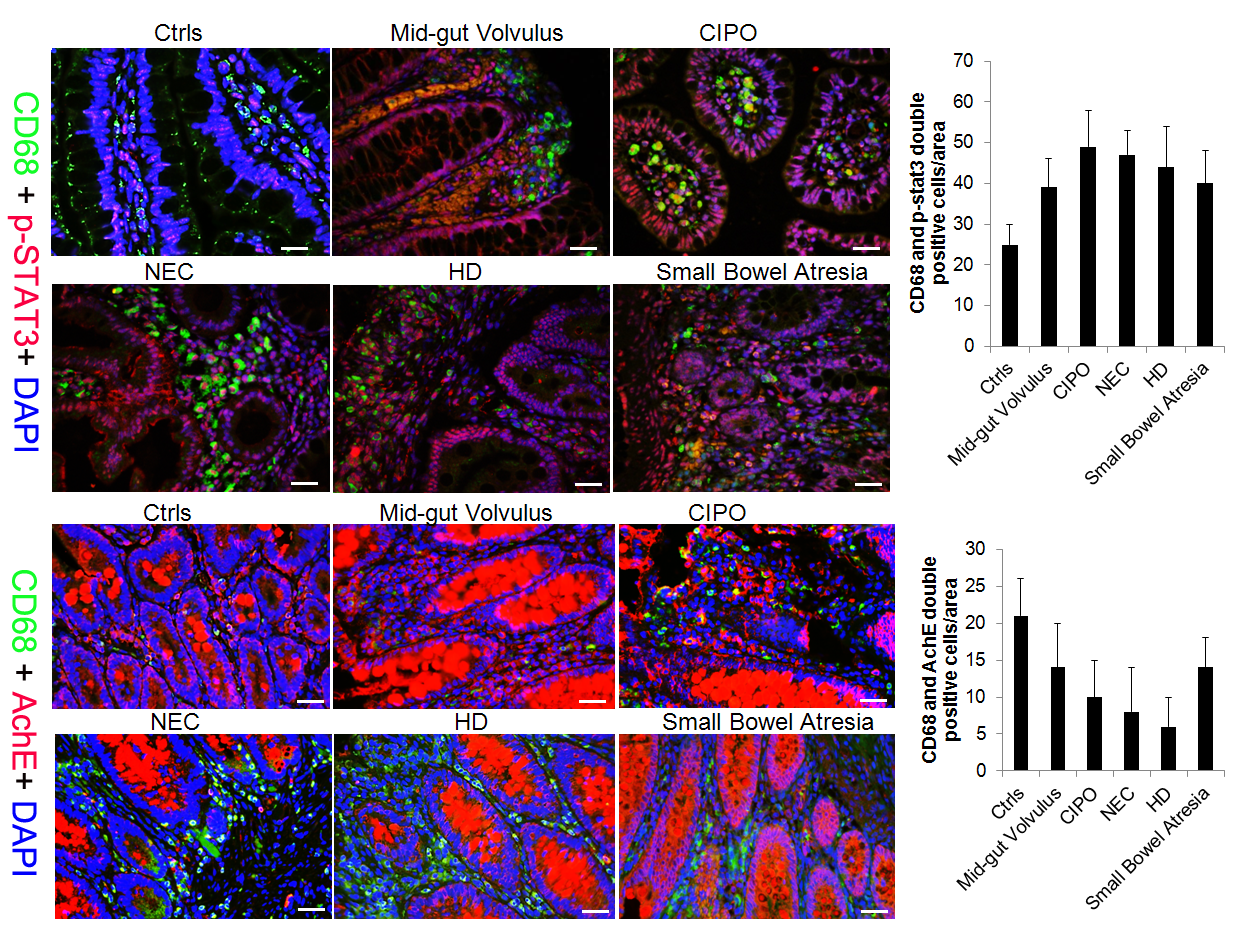
**

**Figure 5. The expression of p-STAT3 and AChE in mice intestinal macrophages of DSS model.** Scale bar = 50 μm.

**
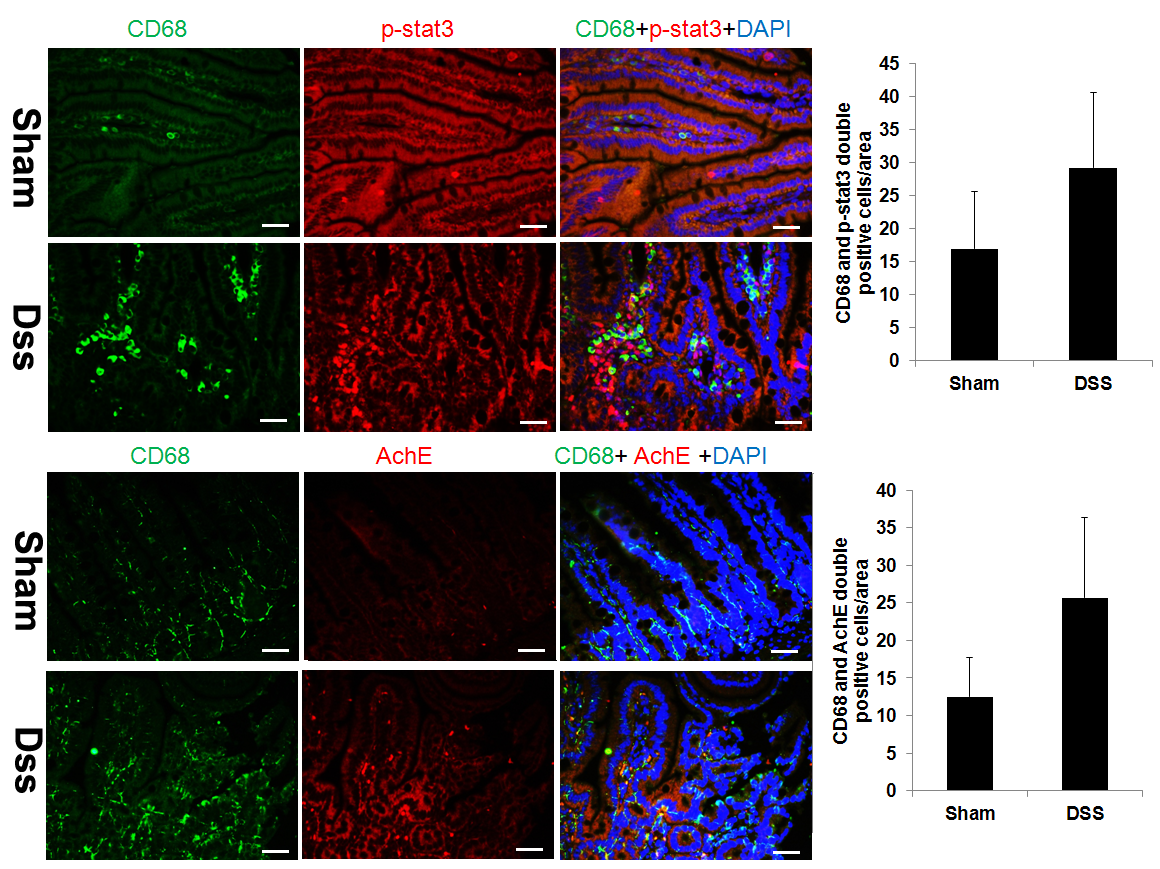
**
